# Risk factors for human papillomavirus infection prevalent among Uyghur women from Xinjiang, China

**DOI:** 10.18632/oncotarget.18901

**Published:** 2017-06-30

**Authors:** Guzhalinuer Abulizi, Hua Li, Patiman Mijiti, Tangnuer Abulimiti, Jing Cai, Jie Gao, Dandan Meng, Reyihanguli Abula, Tunishahan Abudereyimu, Anarguli Aizezi, You Lin Qiao

**Affiliations:** ^1^ 5th Department of Gynecological Surgery, Affiliated Tumor Hospital of Xinjiang Medical University, Ürümqi, Xinjiang Uyghur Autonomous Region, China; ^2^ Cervical Cancer Screening Office, Maternal and Child Health Care Center of Karakax County, Xinjiang Uyghur Autonomous Region, China; ^3^ Department of Cancer Epidemiology, National Cancer Center/Cancer Hospital, Chinese Academy of Medical Sciences and Peking Union Medical College, Beijing, China

**Keywords:** risk factor, human papillomavirus, Uyghur, careHPV, prevalent

## Abstract

We investigated the incidence of HPV and risk factors for infection among rural Uyghur women in the Xinjiang province of northwestern China, where there is a high incidence of cervical cancer. We used the careHPV kit to test 6000 sexually active Uyghur women aged 21 to 60 years for HPV, and conducted a comprehensive questionnaire survey to identify relevant HPV infection factors. Our data show the HPV infection rate to be 8.42%, which is lower than 11.7% reported worldwide, despite the higher cervical cancer incidence. Multivariate logistic regression revealed that Uyghur women that had (a) poor personal hygiene and care; (b) no previous gynecological examination; (c) a higher education level; (d) unprotected sex and inadequate personal hygiene; (e) used their fingers for vaginal cleaning (f) smoking husbands and (j) used sanitary napkins or toilet paper during menstruation or used clod as the bathroom wipe material were at greater risk for HPV infection. This suggests that proper interventions that improve personal hygiene, including not using ones fingers for vaginal cleaning, use of condoms, regular gynecological exams and a reduction in smoking by spouses could lower the cervical cancer risk by lowering HPV infection rates. In addition, increasing awareness among more educated women regarding HPV and implementation of effective interventions could reduce the risk of HPV infection in Uyghur women.

## INTRODUCTION

Human papillomavirus (HPV) infection is a necessary and common risk factor for cervical cancer [1]. In most countries, HPV infection is very common and is dependent on the age and the sexual habits of the populations [2]. In China, the incidence of cervical cancer is greater in remote rural or mountainous areas [3]. The Uyghur is an ethnic group that reside primarily in Xinjiang that is located in northwestern China and have distinct lifestyle, religion, customs and genetic background than other Chinese ethnicities [4]. The Uyghur women have high incidence and mortality due to cervical cancer compared to other ethnic groups [5, 6]. Nearly 80% of cases are diagnosed at an advanced stage that contraindicates surgical intervention. The low social status and the lack of awareness of cervical cancer among Uyghur women of southern Xinjiang combined with lack of systematic screening and preventive measures in this vast rural geographic region have resulted in low rates of early detection, diagnosis and treatment [7–10].

Since cervical cancer is an important public health problem in the Xinjiang Uyghur Autonomous Region of China, we explored the status of HPV infection and the prevalence of risk factors that contribute to high incidence of cervical cancer in Uyghur women.

## RESULTS

### Influence of age, education and occupational criteria on HPV infection rates

**Figure 1:**
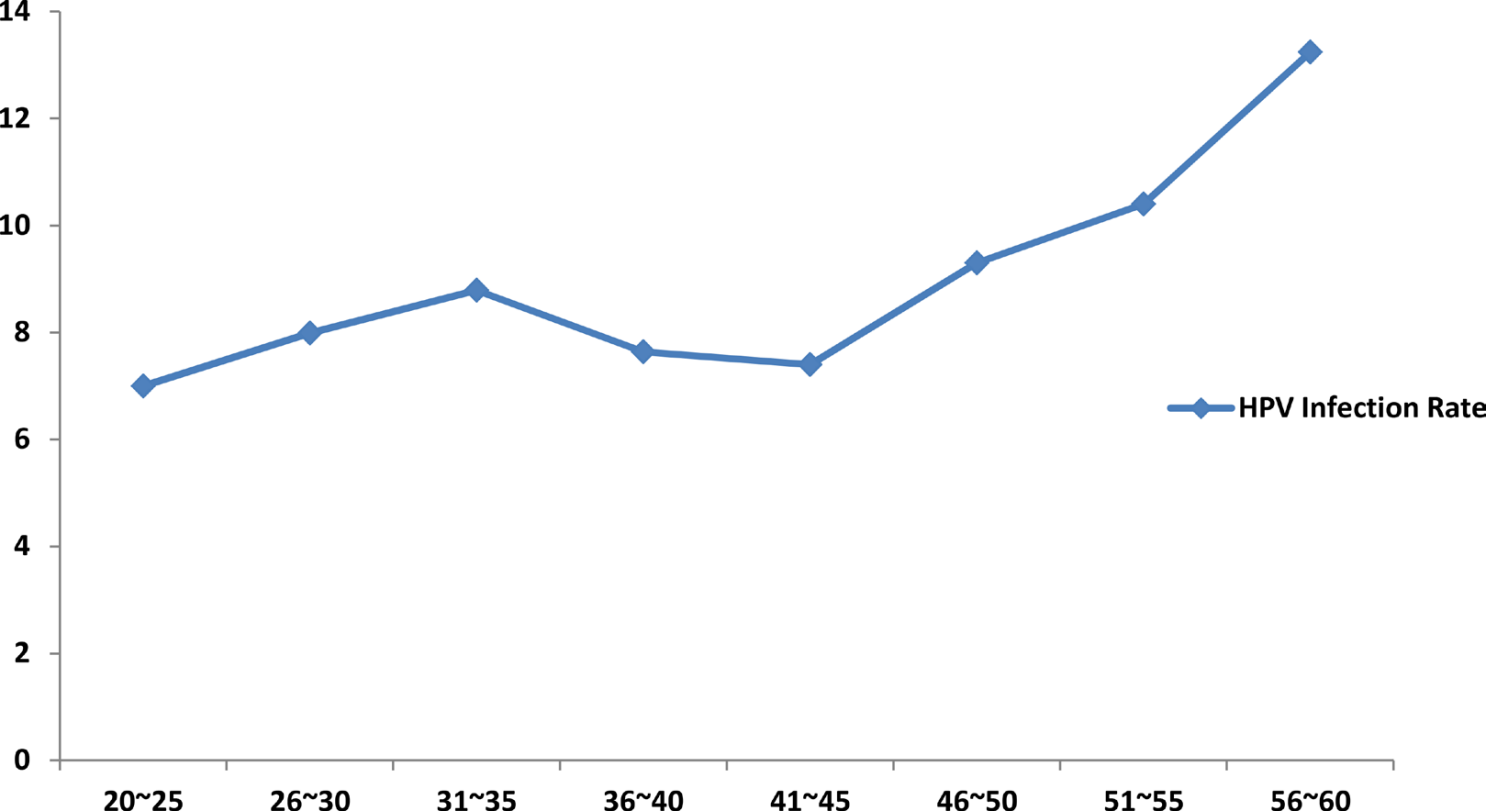
Relation between age and HPV infection rate among Karakax Uyghur women

Among the 6000 participants, 505 were HPV-positive (8.42%) with the mean age of the participants being 38.50 ± 9.71 (21–60) years. When the participants were divided into eight age groups of 5-year increments, those between 41 and 45 years formed the largest group (20.27%), whereas those between 56 and 60 years were the smallest (4.78%). HPV infection rates increased with age from 21 years until 31–35 years of age followed by a gradual decrease with the lowest being the 41–45 age group (Figure 1). Further, the HPV infection rates again increased from 45 yrs onwards and peaked at the age of 60.

In terms of occupation, 93.57% (5614) of total participants were farmers, whereas the numbers for other occupations were small with the “worker” group having only 26 participants. Among the participants, 64.47% and 21.67% had completed primary and secondary education, respectively. With regard to economic status, 40.03% and 31.90% of the participants had annual family incomes in the ranges, ¥5000–10,000 and ¥10,000–30,000 ranges, respectively.

The infection rate among non-farmers (including public servants, factory workers, self-employed women, housewives and other occupations) was higher than that of farmers. Also, higher HPV infection rates were observed among those with higher education including junior college experience or higher degrees (*P* < 0.001). A higher HPV infection rate was observed for participants with a higher family income (> ¥30,000). HPV infection rates was higher in women with family history of cancer, but was not dependent on the cancer types. Women who had never undergone a gynecological examination showed a higher risk of HPV infection (Table 1).

**Table 1:** HPV infection rate of karakax uyghur women based on demographic information

Individual Characteristics	*N* (%)	HPV-positive, *n* (%)	*P*-value
**Occupation**			**< 0.001**
Farmer	5614 (93.6)	450 (8.02)	
Non-farmer	386 (6.4)	55 (14.25)	
**Education level**			**< 0.001**
Illiterate	753 (12.6)	37 (4.91)	
Primary	3748 (62.5)	313 (8.35)	
Secondary	1300 (2.17)	121 (9.31)	
Junior college and above	199 (3.3)	34 (17.09)	
**Annual family income (¥)**			**0.018**
< 5000	811 (13.5)	40 (4.93)	
5000–10,000	2402 (40.0)	146 (6.08)	
10,000–30,000	1914 (31.9)	124 (6.48)	
> 30,000	378 (6.3)	40 (10.58)	
**Family history of cancer**			**0.012**
Yes	322 (5.4)	40 (12.42)	
No	5678 (94.6)	465 (8.19)	
**Type of Cancer with Family History**			0.483
Cervical Cancer	218 (67.7)	29 (13.30)	
Other	104 (32.3)	11 (10.58)	
**Prior gynecological examination**			**<0.001**
Yes	5216 (86.9)	309 (5.92)	
No	784 (13.1)	196 (25.00)	
**Prior cervical smear test**			0.693
Yes	1469 (24.5)	120 (8.17)	
No	4531 (75.5)	385 (8.50)	

### Influence of marriage and child birth conditions on HPV infection rates

The mean age of marriage was 16.43 years (range: 11–29 years). In this study, remarried women had a higher HPV infection rate than those married only once or single (widowed or divorced) women. The mean age of first childbirth was 19.31 years, and the numbers of pregnancies and childbirths were 4.24. The HPV infection rates separated based on the marital and delivery status of the patients is shown in Table 2. Data showed that women who delivered at home had a higher HPV infection rate (8.60%) than those who delivered in a hospital (8.25%). Additionally, higher HPV infection rates were observed in those with a higher number of marriages and with greater frequency of sexual intercourse (*P* < 0.001).

**Table 2:** HPV infection rate of karakax uyghur women based on marrital and delivery information

Individual Characteristics	*N* (%)	HPV-positive *n* (%)	*P*-value
**Marital status**			**0.005**
Married	2574 (42.9)	184 (7.15)	
Divorced	155 (2.6)	8 (5.16)	
Remarried	3061 (51.0)	300 (9.80)	
Widowed	210 (3.5)	13 (6.19)	
**Frequency of intercourse**			**< 0.001**
< 1/week	503 (8.4)	27 (5.37)	
1/week	2868 (47.8)	178 (6.21)	
2–3/week	2524 (42.1)	286 (11.33)	
≥ 4/week	105 (1.8)	14 (13.33)	
**Number of marriages**			0.023
1	2698 (45.0)	194 (7.19)	
2–3	2913 (48.5)	270 (9.27)	
> 3	389 (6.5)	41 (10.54)	
**Age at first childbirth (yrs)**			0.131
≤ 15	266 (4.6)	32 (12.03)	
16–20	3161 (55.0)	264 (8.35)	
> 20	2322 (40.4)	192 (8.27)	
**Number of pregnancies**			0.128
≤ 3	2736 (45.6)	214 (7.82)	
> 3	3264 (54.4)	291 (8.92)	
**Number of childbirths**			0.683
≤ 3	3923 (65.4)	326 (8.31)	
> 3	2077 (34.6)	179 (8.62)	
**Contraceptive method**			
Intrauterine device	3738 (83.0)	288 (7.70)	**0.011**
Sterilization	493 (10.9)	53 (10.75)	**0.040**
Oral contraceptive	499 (11.1%)	25 (5.01%)	**0.002**
Condom	454 (10.1%)	32 (7.05%)	0.263
Others	36 (0.8%)	5 (13.89%)	0.274
**Mode of delivery**			0.248
Eutocia	5335 (88.9%)	452 (8.47%)	
Dystocia	414 (6.9%)	29 (7.00%)	
**Location of delivery**			**0.001**
Home	3326 (55.4%)	286 (8.60%)	
Hospital	1381 (23.0%)	114 (8.25%)	

### Influence of birth control methodology, personal hygiene and partner profile on HPV infection rates

Out of 6000 participants, 4503 used contraceptives (75%), whereas 367 of the 505 HPV-positive participants had used contraceptives. The use of contraceptives did not alter the rate of HPV infection. Nearly 83.01% of the women chose intra-uterine devices, whereas those using oral contraceptives, sterilization and condoms were 11.08%, 10.95%, and 10.08%, respectively. Since participants could choose multiple options in this category, the sum of the percentages of different contraceptive methods exceeded 100%. The results showed that women who underwent sterilization exhibited the highest rate of HPV infection (10.75%) followed by women using intra-uterine devices (7.70%) and oral contraceptives (5.01%) and the differences among the three were significant (*P* ≤ 0.05). Table 3 shows the differences in HPV infection rates based on personal hygiene conditions. Our study showed that HPV infection rates were higher among participants that used sanitary napkins or toilet paper than among those using regular cloths. In addition, women using clod in the bathroom showed higher infection rates than those using toilet paper or other paper products. In summary, lower personal care scores were associated with higher HPV infection rates. In addition, participants who showered frequently and those who changed their underpants frequently had lower HPV infection rates. Women who used their fingers for vaginal cleaning before and after intercourse and before performing a prayer displayed higher infection rates than those who did not. Vaginal cleaning was analyzed separately because in Islam it is believed that semen should be washed away after sexual intercourse, and Uyghur women typically washed their vaginas by putting their fingers into vagina and used no instrument or detergent.

**Table 3:** HPV infection rate of karakax uyghur women based on personal hygiene habits

Personal hygiene condition	*N* (%)	HPV-positive, *n* (%)	*P*-value
**Underpants Change Frequency (days between change)**			**< 0.001**
1–5	4382 (73.0)	346 (7.90)	
7	1554 (25.9)	148 (9.52)	
≥ 14	64 (1.1)	11 (17.19)	
**Is vagina washed?**			**0.003**
Yes	3299 (55.0)	309 (9.37)	
No	2701 (45.0)	196 (7.26)	
**Bathroom wipe material**			**< 0.001**
Clod	4468 (74.5)	400 (8.95)	
Paper and others	1532 (25.5)	105 (6.85)	
**Menstrual product**			**< 0.001**
Sanitary napkin or toilet paper	4935 (82.3)	476 (9.65)	
Cloth /paper	1065 (17.7)	29 (2.72)	
**Undergarment change**			0.293
Yes	1164 (19.4)	81 (6.96)	
No	4836 (80.6)	424 (8.77)	
**Shower frequency (days between showers)**			**< 0.001**
1–3	1482 (24.7)	79 (5.33)	
3–5	1913 (31.9)	187 (9.78)	
7–14	2564 (42.7)	266 (10.37)	
≥ 1 month/wash	41 (0.7%)	7 (17.07)	
**Score for Personal care**			**< 0.001**
0–2	3450 (57.5)	355 (10.29)	
3–4	2550 (42.5)	150 (5.88)	

Women with non-farmer husbands with higher education exhibited a higher rate of HPV infection. In addition, 120 of the 1139 participants (18.98% of total) with partners who had smoking history were HPV-positive and this was significantly higher compared to the infection rate of participants with non-smoking partners. However, there was no association between HPV infection rates and the smoking location (*P* = 0.100) or drug abuse history of the partner (*P* = 0.452) (Table 4).

**Table 4:** HPV infection rate of karakax uyghur women based on partner's characteristics

Partner ‘s Characteristics	*N* (%)	HPV-positive, *n* (%)	*P*-value
**Occupation**			**0.001**
Farmer	5381 (89.7)	430 (7.99)	
Non-farmer	619 (10.3)	75 (12.12)	
**Education level**			**0.002**
Illiterate	607 (10.1)	50 (8.24)	
Primary	3688 (61.5)	285 (7.73)	
Secondary	1489 (24.8)	136 (9.13)	
Diploma and above	216 (3.6)	34 (15.74)	
**Number of marriages**			0.881
1	2618 (43.6)	181 (6.91)	
2–3	2583 (43.1)	240 (9.29)	
> 3	799 (13.3)	84 (10.51)	
**Smoker**			**< 0.001**
Yes	1139 (20.7%)	120 (10.54)	
No	4356 (79.3%)	385 (8.84)	
**Smoking location**			0.100
Indoor	504 (44.2)	42 (8.33)	
Outdoor	635 (55.8)	28 (4.41)	
**Drug abuse**			0.452
Yes	23 (0.4)	1 (4.35)	
No	5472 (99.6)	504 (9.21)	

### Analysis of high risk factors for HPV infection in the Uyghur women

The univariate analysis identified that the factors associated with HPV infection included age, marital status, occupation, education level, family history of cancer, location of child birth, number of marriages, intercourse frequency, use of intra-uterine devices, use of oral contraceptives, menstrual product choice, choice of bathroom wipe material, personal care score, Cleaned vagina with fingers, previous gynecological examination and if the husband was a smoker. The multivariate logistic regression analysis showed that the women with the following characteristics were significantly associated with HPV infection (*P* < 0.05): (a) women with long interval between showers (*OR = 4.358*); (b) no previous gynecological examination (*OR = 4.107*); (c) with junior college education and above (*OR = 3.369*); had frequent intercourse (*OR = 2.222*); used oral contraceptives (*OR = 2.071*); changed underwear less frequently (*OR = 1.620*); cleaned their vaginas with fingers (*OR = 1.608*); used intra-uterine devices (*OR = 1.467*); whose husbands were smokers (*OR = 1.377*); used sanitary napkin or toilet paper for menstrual product (*OR = 0.584*) and chose clod as bathroom wipe material (*OR = 0.402*) as shown in Table 5.

**Table 5:** Logistic regression model for risk factors of hpv infection in karakax uyghur women

Variable	Regression coefficient	Standard error	Wald χ2 value	P-value	Odds ratio	95% confidence interval	
						Lower limit	Upper limit
Education level			14.215	0.003			
Primary	0.422	0.242	3.030	0.082	1.524	0.948	2.450
Secondary	0.376	0.267	1.980	0.159	1.456	0.863	2.457
College and above	1.215	0.335	13.182	0.000	**3.369**	1.749	6.491
Intercourse frequency			11.859	0.008			
1 ×/week	0.374	0.308	1.477	0.224	1.454	0.795	2.660
2–3 ×/week	0.799	0.313	6.508	0.011	**2.222**	1.203	4.104
≥ 4 ×/week	0.477	0.538	0.784	0.376	1.611	0.561	4.628
Intra-uterine device	0.383	0.136	7.967	0.005	**1.467**	1.124	1.915
Oral contraceptive	0.728	0.309	5.538	0.019	**2.071**	1.129	3.796
Menstrual product	–0.538	0.239	5.072	0.024	**0.584**	0.366	0.933
Bathroom wipe material			11.936	0.003			
Clod	–0.911	0.264	11.936	0.001	**0.402**	0.240	0.674
Others	–18.471	4034.30	0.000	0.996	0.000	0.000	.
Vagina wash	0.45	0.130	13.340	0.000	**1.608**	1.246	2.075
Shower frequency			8.463	0.037			
3–5 days	0.137	0.161	0.726	0.394	1.147	0.837	1.572
7–14 days	–0.137	0.188	0.530	0.467	0.872	0.603	1.261
≥ 1 month	1.472	0.647	5.175	0.023	**4.358**	1.226	15.489
Underwear change frequency			17.301	0.000			
1–7 days	–0.811	0.203	16.027	0.000	0.444	0.299	0.661
≥ 14 days	0.483	0.589	0.672	0.412	**1.620**	0.511	5.137
Prior gynecological examination	1.413	0.142	99.303	0.000	**4.107**	3.111	5.423
Partner smoking	0.320	0.177	3.280	0.070	**1.377**	0.974	1.947
Constant	–4.412	0.522	71.487	0.000	0.012		

## DISCUSSION

Previous surveys of about 100,000 women across five continents had shown that the HPV positive rate is 11.7% (range: 6.1% to 33.5%) with normal cytology [11, 12]. The HPV infection rate in the current study was lower than the average reported in other countries [13–15] and in other areas in China (18.0%) [3, 16]. The reason for the low incidence of HPV infection and high incidence of cervical cancer among Uyghur women remains unclear. A fuller understanding of the immunological basis for HPV pathogenicity may shed light on this issue. In our study, the HPV infection rate of women older than 45 years was significantly higher and in agreement with previous reports [17, 18]. However, our study showed that HPV infection rates among women with better education, higher family income, non-farming background (self and partner) was significantly higher than those who were farmers, lowly educated with lower income. This was contradictory to previous studies and suggested that Uyghur women with higher education and economic levels assumed a more open attitude towards sex [19, 17, 20]. Also, our finding that remarried women had higher HPV infection rates than single women was in agreement with a previous report by Shi and colleagues [17], but, contrary to a report by Zhao and colleagues [19].

Previously, having multiple sexual partners was reported as an independent predictor of both prevalent and incident HPV infection [17, 21, 22]. In the present survey less than 10 out of 6000 participants admitted to having extramarital sexual partners and therefore the question was excluded from statistical analysis. The reasons include the fact that the Uyghur's practice Islam that prohibits extramarital sexual partners and regard the extramarital love as a serious offence. Also, the low social status of Uyghur women potentially resulted in instant divorce if caught having extramarital affairs.

Our study also showed that in the Uyghur women, age of first childbirth or first intercourse was not associated with HPV infection contradicting a previous study that showed women experiencing first childbirth or first intercourse at a young age was at risk for cervical pathological changes [23]. One possible reason could be that nearly 93.1% women in our study were married before 20 years of age and therefore the differences in HPV infection rates were not apparent. Also, women delivering at home or married more than twice or with higher frequency of intercourse showed higher HPV infection rates. However, there was no association with different modes and frequencies of child delivery. This was contradictory to previous reports that showed vaginal delivery as a risk factor for vaginal and cervical cancer [13, 24].

Regarding modes of contraception, the highest HPV infection rate was found in women that underwent sterilization, followed by women using intra-uterine devices and oral contraceptives. HPV infection rates in women who used condoms were lower than those using intra-uterine devices. The role of intra-uterine devices in reducing the risk of cervical cancer remains controversial [23, 25] and our study could not draw a definite conclusion regarding the effects of intra-uterine devices on HPV infection.

Further, we found that women using sanitary napkin or toilet paper during menstruation had higher HPV infection rate than those using regular cloth. This could be attributed to low quality paper products as well as the fact that the cloth was made of cotton and was washed and sanitized by sun-drying after each use. In addition, higher HPV infection was reported in women using clods since they increased the possibility of infections in the perianal area. Further, women who washed their vaginas with their fingers showed higher HPV infection rates in agreement with a previous Turkish study [26, 27]. Also, HPV infection rates increased with lower personal care scores, showering frequencies, and frequent underpants changes indicating that personal hygiene was associated with HPV infection risk. Another aspect highlighted by our study was that participants with smoking partners showed higher HPV infection rates suggesting that passive smoking was related to HPV infection.

The multivariate analysis confirmed that Uyghur women in Karakax that had greater risk for HPV infection were those that (a) had poor personal hygiene and care; (b) never had gynecological examination previously; (c) had higher education level; (d) were more sexually active without adequate hygiene like less frequent shower and underpants change or washed vagina with their fingers and used intra-uterine devices and oral contraceptives; (e) had smoking husbands and (f) used sanitary napkin or toilet paper for menstrual product or chose clod for bathroom wipe material. Also our findings suggest that proper interventions that improve poor hygiene, use of condoms, regular gynecological exams and quitting smoking of spouses can significantly lower cervical cancer risk. Also, increasing awareness among higher educated women regarding HPV and allowing accurate and effective interventions can reduce the risk of HPV infections.

## MATERIALS AND METHODS

### Patient details, HPV screening test and sample collection

6000 women were recruited for this study from the Karakax County (Karakax town and Zawa, Yawa, Karsay and Kuiya villages) of the Hotan Prefecture (Xinjiang, China). All the participants provided informed consent. The eligible women were aged between 21–60 years, sexually active, not pregnant and had no history of diagnosed CIN, cervical cancer or hysterectomy.

The careHPV^TM^ test (Qiagen Inc., Gaithersburg, MD, USA) was adopted in this study for rapid HPV screening as it is suitable for developing regions and well established in China. This test is based on the leading HPV DNA detection technique, can detect 14 high-risk HPV subtypes that are associated with cervical cancer (16, 18, 31, 33, 35, 39, 45, 51, 52, 56, 58, 59, 66, and 68) in micro-plates by utilizing a patented mixed cocktail probe of 8000-base RNAs, nucleic acid hybridization and amplification followed by detection with chemiluminescence. The careHPV detection technology has been approved by the European Conformity (CE) and the China Food and Drug Administration (CFDA) with the samples testing positive in the careHPV test being considered HPV-positive.

Samples were collected by brushing the cervix 3–5 times in a clockwise circular motion with a sample careHPV collection brush and stored at room temperature in a storage solution. The laboratory detection of careHPV test was performed by the well-trained postgraduate medical students from Affiliated Tumor Hospital of Xinjiang Medical University onsite (Karakax Maternal and child health care hospital) within a day of sample collection.

### Risk factor analysis questionnaire and interview criteria

The research was approved by the Ethics Committee of the Xinjiang Medical University Affiliated Tumor Hospital. The questionnaire design was guided by Prof. Fanghui Zhao [3, 19, 28]. The questionnaire investigated cervical cancer-related factors based on available international and domestic literature and had 70 questions. The interviewing staff included Uyghur Masters female students who were proficient in both Uyghur and Chinese. They were trained for two days prior to the study and taught to avoid using leading questions and alerted about other factors that could affect the accuracy of the survey due to lack of understanding of the questions. The staff was evaluated based on mock interviews performed on hospital patients. A quality-control staff member regularly supervised the quality of the survey on-site. 6000 out of 6504 questionnaires were selected for final data analysis.

Individual participants were interviewed face-to-face. As the questionnaire was in Chinese, the interviewing staff needed to translate and explain the content of the questionnaire that consisted of closed-ended questions. Apart from the questions requiring specific values (age of participant, age at marriage, age at first childbirth, number of marriages, pregnancies and childbirths and the smoking history of the participant's partner), the remaining ones were multiple-choice from which the participants could choose one or more relevant answers.

Regarding personal care, there were four questions, namely, (a) if the vulva was washed before intercourse, (b) if the hands were washed before washing the vulva, (c) if the vulva was washed after intercourse, and (d) if shower was taken after intercourse. The participants received 1 point for every “Yes” and 0 points for every “No”. The total score for this section ranged from 0 to 4 points, with higher scores indicating better personal care. Another set of questions were regarding if the vagina was washed before and after the intercourse and if the vagina was washed before performing a prayer. Since there were three variables, the participants received 1 point for every “Yes” and 0 point for every “No” and therefore, the total score for this segment ranged from 0 to 3 points.

### Statistical analysis

Data entry and management was performed using the Epidata 3.1 software (Odense, Denmark). Data processing and statistical analyses were performed using the SPSS19.0 software (IBM Corp., Armonk, NY, USA). The basic characteristics and the quantitative data of the participants were presented as mean ± standard deviation (SD), whereas the qualitative data were presented as absolute frequencies or frequencies. The χ^2^ test was used to evaluate the differences in HPV infection rates based on factors including individual characteristics, marital and parental status, contraceptive measures, personal hygiene and partner conditions. Multivariate analyses were performed to analyze the effects of the investigated factors on HPV infection rates using dichotomous logistic regression. The logistic regression model was established using a stepwise method to select variables that showed statistical significance, with α = 0.05 for inclusion and α = 0.10 for exclusion. Statistical significance was defined as *P* ≤ 0.05. All variables were analyzed together in the multivariate analyses.
